# From trait emotional intelligence to wellbeing and growth: the role of adaptive coping during hardship

**DOI:** 10.3389/fpsyg.2026.1924156

**Published:** 2026-09-15

**Authors:** Maria-Jose Sanchez-Ruiz, Myriam El Khoury-Malhame, Roni Chaaya, Chloe Mechleb, Ramón-Iker Soria-Royuela, Rita Doumit

**Affiliations:** 1 Department of Personality Psychology, Psychological Assessment and Treatment, School of Psychology, Universidad Nacional De Educacion a Distancia, Madrid, Spain; 2 Department of Social and Education Sciences, School of Arts and Sciences, Lebanese American University, Beirut, Lebanon; 3 Suliman S. Olayan School of Business, American University of Beirut, Beirut, Lebanon; 4 Science, Technology and Society Research Unit, Centro De Investigaciones Energeticas, Medioambientales y Tecnologicas, Madrid, Spain; 5 Alice Ramez Chagoury School of Nursing, Lebanese American University, Byblos, Lebanon

**Keywords:** coping strategies, emotional competence, post-traumatic growth, psychological wellbeing, trait emotional intelligence

## Abstract

**Introduction:**

Adolescence, a critical stage of development, requires robust emotional competences to manage psychological distress. This study examines how trait Emotional Intelligence (trait EI) relates to adolescent wellbeing and post-traumatic growth in an adverse sociopolitical context.

**Methods:**

The study included 766 adolescents (417 female) aged 14–20 years (*M* = 16.34, *SD* = 1.18) from Lebanese public schools. Measures assessed trait EI through the profile of emotional competences (PEC), adaptive coping (Brief-COPE), wellbeing (WHO-5), and post-traumatic growth (PTGI-SF). Structural equation modeling (SEM) was performed with robust estimators.

**Results:**

The data supported the model, demonstrating a direct positive association between intrapersonal EI and adaptive coping (β = 0.48, *p* < 0.001) and interpersonal EI and adaptive coping (β = 0.34, *p* < 0.001), and between adaptive coping and both wellbeing (β = 0.47, *p* < 0.001) and post-traumatic growth (β = 0.73, *p* < 0.001). Adaptive coping showed significant indirect associations linking intrapersonal EI with wellbeing (β = 0.22, *p* < 0.001) and post-traumatic growth (β = 0.35, *p* < 0.001), as well as the association of interpersonal EI with wellbeing (β = 0.16, *p* < 0.001) and post-traumatic growth (β = 0.25, *p* = < 0.001).

**Discussion:**

Findings highlight that adaptive coping strategies (i.e., active coping, positive reframing, planning, and acceptance) are plausible coping-related pathways linking trait EI to wellbeing and post-traumatic growth, stressing the importance of integrating these positive psychological dispositions into wellbeing programs and interventions targeting adolescents facing adversity.

## Introduction

Adolescence is a developmental stage marked by substantial neurobiological, emotional, and social transformations that increase vulnerability to stress-related disorders (O’Donnell, 2024). In Lebanon, cumulative exposure to political instability, economic collapse, social unrest, and infrastructural breakdown elevates the risk of psychological distress among youth (Nasr et al., 2024; Al-Khalil et al., 2025). Given these surrounding circumstances, there is an urgent need to identify individual-level protective factors that can support youths’ adaptive functioning under chronic adversity.

One such factor is trait Emotional Intelligence (EI), a constellation of emotional perceptions assessed through questionnaires and rating scales (Petrides et al., 2007), which has shown to predict a myriad of life outcomes such as academic engagement and performance, physical and mental health, life and relationship satisfaction, and wellbeing indicators, among others (Petrides et al., 2016). The Profile of Emotional Competence (PEC) offers a detailed assessment of trait EI, distinguishing between competences applied to one’s own emotions and those applied to the emotions of others (Brasseur et al., 2013). These competences consist of recognition, understanding, and regulation of personal emotional experiences, as well as interpersonal emotional competence, which involves perceiving, responding to, and managing the emotions of others (Brasseur et al., 2013).

Among adolescents, trait EI plays a crucial role in managing stress, primarily by enhancing emotional regulation and facilitating adaptive responses to emotional challenges (Mancini et al., 2024). High trait EI-scoring adolescents are more likely to engage in effective forms of coping (Arora and Kumar, 2023), which help them better navigate stress and adopt coping strategies that are more adaptive and less avoidant (Zhylin et al., 2024). Coping strategies, as cognitive and behavioral tools employed to manage demands from stressful situations, have been broadly classified as adaptive or maladaptive (Bellizzi and Blank, 2006; Mugavero et al., 2009). Maladaptive coping, such as avoidance, denial, self-blame, and social withdrawal, may offer short-term relief but often exacerbates long-term psychological issues, including anxiety, depression, and post-traumatic stress (Compas et al., 2017). Adaptive coping strategies such as problem-solving, cognitive reframing, and seeking social support foster psychological resilience by reducing stress and supporting long-term wellbeing (Augusto-Landa et al., 2024).

During adolescence, a period marked by frequent exposure to academic, social, and identity-related stressors, higher levels of trait EI have been consistently linked to reduced symptoms of anxiety and depression (Gardner and Lambert, 2019), enhanced stress management (Arora and Kumar, 2023), and improved emotional regulation (Mancini et al., 2024). One explanation of these links is that trait EI plays a critical role in shaping adolescents’ coping behaviors. Research suggests that trait EI supports adaptive coping in youth, likely by promoting emotional awareness and behavioral flexibility, enabling adolescents to respond more effectively to challenges (Du Plessis, 2023) and sustain emotional balance (Costa and Faria, 2025).

Additionally, trait EI facilitates the selection of coping strategies aligned with emotional needs and situational demands, thereby fostering greater resilience in the face of stress (Sánchez-Álvarez et al., 2016). During challenging times, such as the COVID-19 pandemic, individuals with high trait EI were more likely to engage in adaptive coping, which, in turn, related to lower psychological distress and higher levels of wellbeing (Sanchez-Ruiz et al., 2021b). Together, these findings suggest that trait EI may foster adaptive coping behaviors essential to psychological wellbeing in challenging circumstances, support recovery, and lead to positive transformation.

### Trait EI, coping, and post-traumatic growth

While trauma exposure often results in symptoms of post-traumatic stress (PTS), it can also give rise to post-traumatic growth (PTG), defined as positive psychological change that follows the struggle with highly challenging life circumstances (Shakespeare-Finch and Lurie-Beck, 2014). These changes may include a renewed appreciation of life, personal strength, and spiritual development (Taku et al., 2008). PTG emerges through intentional reflection and deriving meaning, as individuals reframe traumatic experiences and revise their understanding of the self, others, and the world (Park, 2010).

Psychological responses to trauma are diverse, with PTS and PTG often co-occurring or emerging sequentially rather than representing mutually exclusive outcomes (Doumit et al., 2025). Such variability may be shaped by underlying emotional mechanisms such as trait EI, which may serve as a protective psychological resource, shaping how individuals manage emotional turmoil while facilitating cognitive restructuring and growth-oriented reflection. An association between trait EI and PTG has been identified in a sample comprising individuals who have experienced a traumatic event (Tuck and Patlamazoglou, 2019). Similarly, more recent evidence supports this pathway. For instance, Cannavò et al. (2024) found that individuals with higher trait EI report greater PTG, higher life satisfaction, and lower emotional distress following trauma (Cannavò et al., 2024).

Individuals with higher trait EI are better equipped to engage in effective coping strategies, regulate emotional responses, and reappraise traumatic events in constructive ways and perceive adversity as a challenge rather than a threat; thus, trait EI could function both as a buffer against suffering and a catalyst for psychological transformation (Sanchez-Ruiz et al., 2021b). Thus, adaptive coping could act as the mechanism through which trait EI influences psychological outcomes, buffering PTS symptoms while enabling meaning-making that promotes PTG.

### The Lebanese context and the present study

Lebanese adolescents face a unique convergence of risks marked by a series of compounded stressors and their consequences, including the socio-economic collapse, political instability, and the earlier global COVID-19 and Beirut port explosion. This unstable sociocultural context heightens their psychological vulnerability and amplifies the need for interventions that foster emotional competences (Buchlmayer et al., 2023). This study draws on data from a broader initiative titled *Yes to Emotions in Youth (YEY)*, an empirically grounded, culturally adapted, and youth-led emotional intelligence program developed in response to Lebanon’s ongoing crises. The program has shown preliminary effectiveness in decreasing psychopathology and increasing wellbeing (see Sanchez-Ruiz et al., 2026, for more details).

The novelty of the present study lies in its integrative exploration of how intrapersonal and interpersonal emotional competences contribute to adolescent wellbeing and post-traumatic growth (PTG) through adaptive coping mechanisms within a uniquely strained sociocultural environment. Unlike most prior research conducted in Western or stable contexts, this study situates emotional functioning within the chronic adversity experienced by Lebanese youth, an understudied population repeatedly exposed to economic collapse, political instability, and collective trauma.

Existing research on trait Emotional Intelligence (EI) in Lebanese samples has demonstrated its protective role against psychopathology and its positive association with wellbeing (e.g., Khalaf et al., 2026). The construct has also shown robust psychometric properties in Lebanon, supporting its cross-cultural validity and applicability in this context (Sanchez-Ruiz et al., 2021a). In addition, trait EI has been linked to lower levels of disordered eating (Sanchez-Ruiz et al., 2019) and higher levels of adaptive coping, mindfulness, and self-care (Sanchez-Ruiz et al., 2024). However, despite these valuable insights, the psychological mechanisms through which trait EI influences wellbeing and growth remain underexplored, particularly in non-Western, resource-constrained contexts.

To address this gap, the present study examines adaptive coping—specifically strategies such as positive reframing, acceptance, planning, and active coping—as a potential mechanism through which emotional competence may contribute to both wellbeing and posttraumatic growth (PTG). By testing a structural equation model (SEM) in a large sample of adolescents facing prolonged hardship, this study provides novel empirical evidence for how emotional competences can translate into resilience and growth through adaptive coping.

Based on the reviewed literature, five hypotheses are proposed:

(H1) Intrapersonal and Interpersonal emotional competence will be positively associated with adaptive coping strategies.
(H2) Adaptive coping strategies will be positively associated with wellbeing.
(H3) Adaptive coping strategies will be positively associated with PTG.
(H4) Intrapersonal and Interpersonal emotional competence will show significant indirect associations with wellbeing through adaptive coping.
(H5) Intrapersonal and Interpersonal emotional competence will show significant indirect associations with PTG through adaptive coping.

## Materials and methods

### Participants

The sample consisted of 766 high school students aged between 14 and 20 (*M* = 16.34, SD = 1.18), who were recruited from three public schools in Lebanon.

The age distribution was as follows: 14 to 15 years old, 22.72% (*N* = 174), 16 to 17 years old, 57.05% (*N* = 437), over 18 years old, 13.32% (*N* = 102), and 6.92% of the sample had missing information on this variable (*N* = 53). The 13.32% of our sample (over 18 years old) is older than typical for secondary schools in Lebanon. This demographic shift might be attributable to the severe 2019-2020 economic crisis and subsequent monetary inflation in Lebanon. The financial strain led many students to drop out of high-tuition private schools and enroll in the nearly free public school system instead.

About 54.44 % were female (*N* = 417), 42.04% were male (*N* = 322), 1.17% declared themselves from Other gender (*N* = 9), and 2.35% had missing gender information (*N* = 18). The job status of 86% (*N* = 658) of the participants’ fathers was employed, 8% (*N* = 61) unemployed, and 6% (*N* = 47) had missing information on this variable. On the other hand, 67% (*N* = 515) of the participants’ mothers were unemployed, 30% (*N* = 226) were employed, and 3% (*N* = 25) had missing information on the mother’s job status. Financial difficulty was measured on a scale ranging from 0 (no difficulty) to 10 (extreme difficulty), with participants reporting an average score of 5.2 (SD = 1.95). Importantly, the majority of respondents (72.84 %) indicated experiencing at least a moderate to severe level of difficulty in paying their household bills.

### Procedure

The study was part of a larger project Yes to Emotions in Youth (YEY): An Emotional Intelligence Training for Vulnerable Youth in Lebanon, funded by [Masked for Blind Review]. The project employed a quasi-experimental (pre/post-test) mixed-method research design and received administrative approval from the Ministry of Education and Higher Education. The study was also approved by the Institutional Review Board of the authors’ institution [Masked for Blind Review]. After participants and their parents signed the informed consent, participants were asked to report some demographic data and then completed the Arabic-language versions of standardized scales for emotional competence, coping, wellbeing, and post-traumatic growth. All Arabic scales were reviewed to ensure item clarity and cultural appropriateness. Data from this participant cohort have previously contributed to psychometric validation studies of the Arabic WHO-5 and PTGI-SF (Doumit et al., 2025, 2026). Those publications examined the psychometric properties of the respective instruments, whereas the present study addresses a distinct substantive research question concerning the cross-sectional associations among emotional competence, adaptive coping, wellbeing, and post-traumatic growth.

### Measures

#### Profile of emotional competence (PEC)

The Profile of Emotional Competence (PEC; Aouani et al., 2019) assesses intrapersonal and interpersonal emotional competences, with each domain encompassing five core skills, namely identification, comprehension, expression, regulation, and utilization of emotions. Participants responded to 50 items (e.g., “As my emotions arise, I don’t understand where they come from”) on a 5-point Likert-type scale ranging from 1 (“The statement does not describe you at all or you never respond like this”) to 5 (“The statement describes you very well or you experience this response very often”). A reduced version of the PEC was used, excluding the utilization of emotions DOMAIN, since it compromised the results conceptually and statistically, a decision aligned with prior research that encountered similar issues and questioned its distinctiveness and reliability (Nozaki et al., 2019). The English version of the PEC demonstrated moderate to excellent internal consistency across its subscales (α = 0.60–0.83; Brasseur et al., 2013), and the Arabic version has also shown strong psychometric properties (Aouani et al., 2019). In our sample, the internal consistency was modest, falling below the conventional threshold of 0.7. Cronbach’s alpha was 0.63, and McDonald’s omega was 0.69 for the global score. Modest reliabilities were also observed for the interpersonal (α = 0.56; ω = 0.60) and intrapersonal (α = 0.60; ω = 0.63) dimensions.

#### Brief coping orientation to problems experienced inventory (brief-COPE)

Coping strategies were assessed using the Brief COPE inventory (Carver, 1997), a self-report measure evaluating strategies for adaptive coping, maladaptive coping, and support-seeking. For the present analysis, we focused on eight items covering four adaptive subscales, namely Active Coping, Planning, Positive Reframing, and Acceptance; for more information, see Supplementary Table 1. This was a theory-driven decision, as the selected subscales have commonly been conceptualized as adaptive coping strategies in studies using the COPE (e.g., Meyer, 2001) and align directly with the study’s focus on adaptive coping as the potential associational pathway linking emotional competencies with wellbeing and post-traumatic growth. Responses were given on a 4-point Likert scale ranging from 1 (“I haven’t been doing this at all”) to 4 (“I’ve been doing this a lot”). An example item for the Active Coping subscale is, “I’ve been taking action to try to make the situation better.” The Brief COPE has been widely used and validated in an Arabic-speaking population (Alghamdi, 2020). In our sample, the scores’ internal consistency was within the acceptable range (α = 0.77; ω α = 0.83).

#### World Health Organization Wellbeing Index (WHO-5)

The World Health Organization’s 5-item Wellbeing Index (WHO-5; Bech and Østergaard, 2005) is a brief self-report measure of emotional wellbeing over the past 2 weeks. Participants rated five items (e.g., “I have felt cheerful and in good spirits”) on a Likert scale from 0 (“At no time”) to 5 (“All of the time”). Independent psychometric support for the Arabic WHO-5 has been reported among Lebanese older adults (Sibai et al., 2009) and in a cross-national sample of young adults from six Arab countries (Fekih-Romdhane et al., 2025). Doumit et al. (2025) subsequently evaluated the Arabic WHO-5 in a larger sample of Lebanese adolescents that included the participants in the present study and reported satisfactory internal consistency (α = 0.80). Because of this sample overlap, the findings reported by Doumit et al. (2025) are interpreted as complementary psychometric evidence rather than as an independent validation or replication. That study focused specifically on the psychometric properties of the WHO-5, whereas the present study examines distinct constructs and hypotheses. In our sample, internal consistency was excellent (α = 0.83; ω = 0.87).

#### Posttraumatic growth inventory - short form (PTGI-SF)

Posttraumatic growth was measured using the Posttraumatic Growth Inventory – Short Form (PTGI-SF; Cann et al., 2010), which assesses positive changes experienced following traumatic events. The scale covers five domains: personal strength, spiritual change, new possibilities, appreciation of life, and relating to others. Participants responded to 10 items on a 6-point Likert scale ranging from 0 (“I did not experience this change”) to 5 (“I experienced this change to a very great degree”). However, based on the psychometric properties of the PTGI-SF in a Lebanese adolescent sample (Doumit et al., 2026) and in line with a recent publication evidencing the similarity of the 10- and 8-item PTGI-SF (Fekih-Romdhane et al., 2024), the inventory was reduced to 8 items in the present study to keep the scale in its shortest form (Supplementary Table 1). This would ensure ease of use for adolescents, especially in combination with other assessments. An example item is, “I established a new path for my life.” The PTGI-SF has demonstrated excellent internal consistency (α = 0.925) and has been translated into Arabic and validated among Arabic-speaking populations (Veronese and Pepe, 2019) and in a separate Lebanese adult community sample in which both the 8- and 10-item versions were evaluated (Fekih-Romdhane et al., 2024). Doumit et al. (2026) subsequently evaluated the 10-item PTGI-SF in a Lebanese adolescent sample that included participants from the present study and reported adequate internal consistency (α = 0.73; ω = 0.73). Because of this sample overlap, those findings are interpreted as complementary psychometric evidence rather than as an independent adolescent validation or replication. In our sample, reliability indices indicate good internal consistency (α = 0.80; ω = 0.84) (Doumit et al., 2026).

### Data analysis

The statistical analyses were conducted using the R Statistical Software v4.3.3 and the packages: readxl, lavaan, QuantPsyc, energy, MVN, semPlot, psych, EnvStats, car, semptools, doParallel, VIM, foreach, and tidyverse. Initial inspection of the data revealed five coding errors, which were corrected by declaring the affected entries as missing observations. An average of 9 missing values per variable was found, addressed via k-nearest neighbors (k-NN) imputation. Descriptive analyses were also performed, extracting the means, standard deviations, and minimum and maximum values of the scales’ items. Additionally, internal reliability was assessed using both Cronbach’s alpha and McDonald’s omega. Next, correlation analyses were conducted using the Pearson correlation coefficient.

Structural equation modeling (SEM) was selected as the most appropriate method for analyzing the complex relationships proposed in the model. Before conducting SEM, we tested the multivariate normality using the energy test for equal distribution and Mardia’s test. Multicollinearity was examined by calculating variance inflation factors (VIFs), using the model variables as predictors and a binary dummy variable as the dependent variable. This method allowed for the detection of strong predictor interrelationships not captured by correlation coefficients alone (Franke, 2010). The inspection of the VIFs for all the scales showed that none of them had values higher than 1.98, still below the suggested VIF threshold of 2.5, which indicates significant collinearity (Johnston et al., 2018), hinting at the lack of multicollinearity in our data.

Given non-normality and sample size, we applied the MLR estimator with Huber-White robust standard errors and the Yuan-Bentler T2-star test statistic. Model fit was evaluated using four indices: Robust CFI and Robust TLI (with a cutoff of 0.95; Hu and Bentler, 1999), and Robust RMSEA and SRMR (with respective thresholds of 0.06 and 0.08; Hooper et al., 2008). The structure of the SEM model was theory-driven, from the direction of the relationships between scales to the variables included as correlates. During the final stage of model fitting, modification indices and standardized residuals were examined to identify potential sources of local misfit. Only after establishing that they were theoretically meaningful and compatible with the conceptual structure of the model were empirical adjustments considered (Kline, 2023). Residual covariances were retained only when the indicators shared content that was not fully represented by their corresponding latent factor. Within the adaptive coping subscale, residual covariances were specified between the two active-coping indicators (1 and 6), the two positive-reframing indicators (10 and 15), and the two acceptance indicators (16 and 18). This reflects the variance specific to each coping strategy within the Brief-COPE subscale. Additionally, residual covariances were specified between active-coping and planning indicators (1 and 12, as well as 6 and 12). This is because planning involves identifying possible actions, whereas active coping reflects the implementation of such actions. Additionally, acceptance indicators were allowed to covary with one planning indicator (16 and 18 with 19) because acknowledging the reality and constraints of an adverse situation can lead to a more realistic consideration of subsequent steps.

Within the PTGI-SF, residual covariances were specified between items 4 and 8 because these indicators share spiritual change content. Residual covariance was also specified between items 2 and 3 because both indicators reflect related aspects of positively reevaluating life and future possibilities following adversity. Finally, residual covariance was specified between the identification and understanding of others’ emotions indicators of the PEC because these competencies represent closely related stages of interpersonal emotional processing.

## Results

### Descriptive statistics

The means and Median Absolute Deviations (MAD) of the study variables are as follows: Intrapersonal emotional competence (range: 2–10) exhibited a moderate average score (*M* = 6.21, MAD = 2.06), indicating a relatively higher level of emotional competence within the sample. Similar values could be seen with Interpersonal emotional competence (range: 2–10), also with a moderate score (*M* = 6.88, MAD = 1.79). Adaptive coping strategies (range: 1–4) showed a mean of 2.81 (MAD = 0.90), reflecting a moderate use of adaptive coping mechanisms. Psychological wellbeing (WHO-5, range: 0–5) had a mean score of 2.08 (MAD = 1.47), suggesting a lower level of psychological wellbeing on average within the sample. Finally, post-traumatic growth (PTGI, range: 0–5) demonstrated a mean of 3.17 (MAD = 1.51), indicating a moderate degree of post-traumatic growth among participants.

### Correlations among the key study variables

Table 1 presents the Pearson correlation coefficients among the study variables: intrapersonal emotional competence, interpersonal emotional competence, adaptive coping, psychological wellbeing, and post-traumatic growth. Intrapersonal emotional competence was positively associated with adaptive coping (*r* = 0.292, *p* < 0.001). It was also positively related to both outcome variables, namely psychological wellbeing (*r* = 0.414, *p* < 0.001) and post-traumatic growth (*r* = 0.305, *p* < 0.001). Interpersonal emotional competence had positive correlations with adaptive coping (*r* = 0.208, *p* < 0.001), psychological wellbeing (*r* = 0.16, *p* < 0.001) and post-traumatic growth (*r* = 0.32, *p* < 0.001). Similarly, adaptive coping showed positive correlations with psychological wellbeing (*r* = 0.274, *p* < 0.001) and post-traumatic growth (*r* = 0.375, *p* < 0.001). Finally, psychological wellbeing and post-traumatic growth were positively correlated (*r* = 0.295, *p* < 0.001) as well as Interpersonal and Intrapersonal emotional competence (*r* = 0.239, *p* < 0.001).

**TABLE 1:** Pearson correlation coefficients of the variables in the model.

		PTGI	Adaptive coping	Interpersonal EC	Intrapersonal EC
WHO-5	1				
PTGI	0.295***	1			
Adaptive coping	0.274***	0.375***	1		
Interpersonal EC	0.16***	0.32***	0.208***	1	
Intrapersonal EC	0.414***	0.305***	0.292***	0.239***	1

***p < 0.001.

### Structural equation modeling (SEM)

#### Measurement model

The measurement model was assessed to evaluate the adequacy of the latent constructs. All items loaded significantly onto their respective factors, with standardized factor loadings ranging from 0.31 to 0.74 (all *p* < 0.001), indicating acceptable item reliability. Specifically, items for adaptive coping demonstrated standardized loadings between 0.38 and 0.56, psychological wellbeing (WHO-5) items ranged from 0.59 to 0.74, Post-Traumatic Growth Index (PTGI) items ranged from 0.40 to 0.68, the Intrapersonal Emotional Competence subscale ranged from 0.38 to 0.60 and the Interpersonal Emotional Competence subscale ranged from 0.31 to 0.66. The fit of the measurement model was χ^2^(385) = 653.9, *p* < 0.001, RMSEA = 0.032 [90% CI:0.027, 0.036], SRMR = 0.041, Robust CFI = 0.945, Robust TLI = 0.938, slightly below the cut-off criteria for the Robust CFI and TLI (Hu and Bentler, 1999), but it was well below the thresholds for the Robust RMSEA and SRMR (Hooper et al., 2008), supporting the adequacy of the latent constructs and allowing for the evaluation of the structural model. Table 2 offers a clear presentation of the measurement model discussed.

**TABLE 2:** Estimates of the emotional competences SEM model.

A. Regression weights							
**Endogenous variables**	**Exogenous variables**	**Estimate**	**95% CI of the estimates**		**Standardized estimates**	***p*-value**	**SMC**
			**Lower**	**Upper**			
Adaptive coping	Interpersonal EC	0.2	0.11	0.29	0.34	< 0.001	0.48
	Intrapersonal EC	0.22	0.15	0.28	0.48	< 0.001	
WHO5	Adaptive coping	0.97	0.69	1.24	0.47	< 0.001	0.22
PTGI	Adaptive coping	1.63	1.3	1.96	0.73	< 0.001	0.53
B. Indirect effects							
**Endogenous variables**	**Antecedent variables**	**Estimate**	**95% CI of the estimates**		**Standardized estimates**	***p*-value**	
			**Lower**	**Upper**			
WHO5	InterpersonalEC	0.19	0.10	0.28	0.16	< 0.001	
PTGI	Interpersonal EC	0.32	0.18	0.47	0.25	< 0.001	
WHO5	Intrapersonal EC	0.21	0.12	0.30	0.22	< 0.001	
PTGI	Intrapersonal EC	0.35	0.23	0.47	0.35	< 0.001	

#### Structural model

The structural model was evaluated using SEM (see Figure 1), and the hypotheses were tested based on the results. The overall fit of the model was acceptable, suggesting that the model adequately represents the data: χ^2^(389) = 738.84, *p* < 0.001, RMSEA = 0.036 [90% CI:0.032, 0.040], SRMR = 0.049, Robust CFI = 0.929, Robust TLI = 0.92.

**FIGURE 1:**
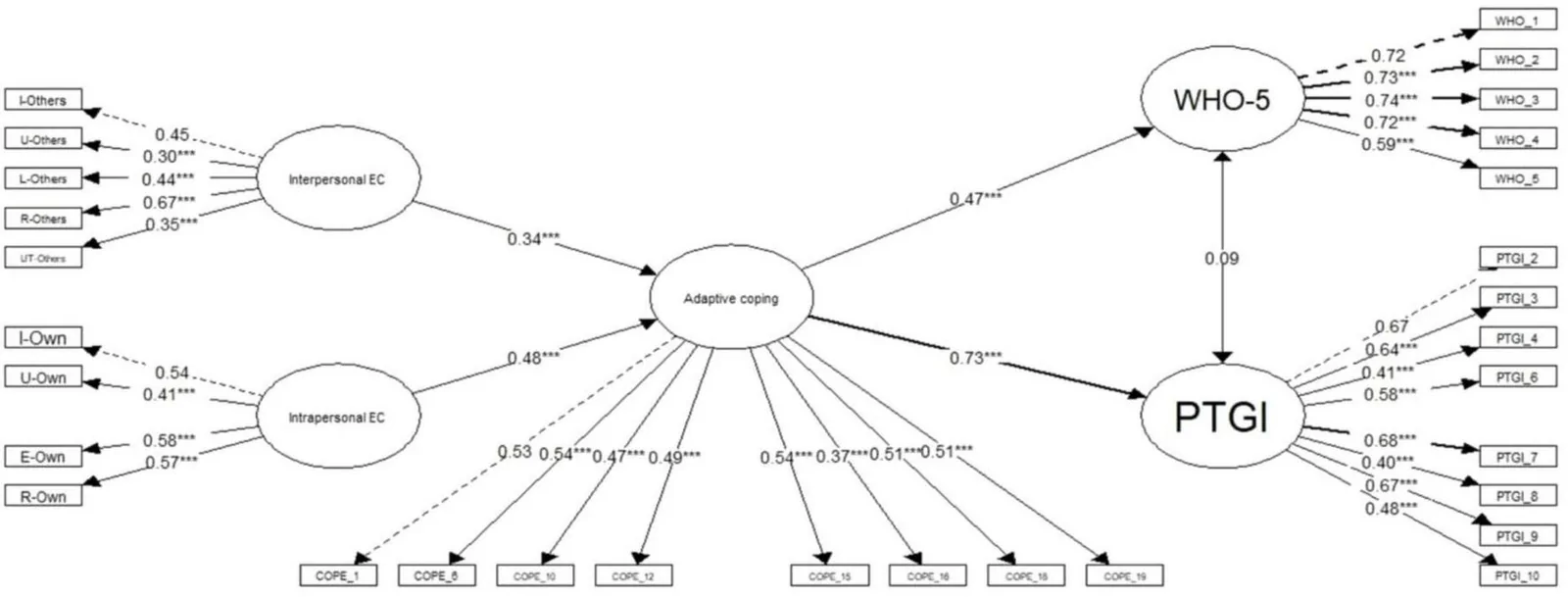
Emotional competence SEM model. ***p < 0.001.

Intrapersonal Emotional Competence was positively associated with adaptive coping (Estimate = 0.22, 95% CI [0.15, 0.28], *p* < 0.001). Interpersonal Emotional Competence was also positively associated with adaptive coping strategies (Estimate = 0.2, 95% CI [0.11, 0.29], *p* < 0.001); see Table 2 for more information on the SEM model. The respective standardized estimates of 0.48 for Intrapersonal Emotional Competence and of 0.34 for Interpersonal Emotional Competence toward adaptive coping indicated a strong positive effect, suggesting that higher levels of emotional competence are associated with better use of adaptive coping strategies. Both emotional competence measures showed a medium strength correlation (*r* = 0.41, *p* < 0.001). Adaptive coping strategies were positively associated with psychological wellbeing (Estimate = 0.97, 95% CI [0.69, 1.24], *p* < 0.001). The standardized estimate of 0.47 indicates a moderate association between adaptive coping and psychological wellbeing. Adaptive coping was also positively associated with post-traumatic growth (Estimate = 1.63, 95% CI [1.3, 1.96], *p* < 0.001). The standardized estimate of 0.73 pointed to the strong positive association between adaptive coping and post-traumatic growth. Also, a non-significant positive correlation between WHO-5 and PTGI (*r* = 0.09, *p* = 0.214) was found.

The indirect-effects analysis indicated that adaptive coping statistically accounted for part of the associations between emotional competence and wellbeing. Significant indirect associations were observed from Interpersonal Emotional Competence (Estimate = 0.19, 95% CI [0.10, 0.28], *p* < 0.001) and from Intrapersonal Emotional Competence (Estimate = 0.21, 95% CI [0.12, 0.30], *p* < 0.001). The standardized estimates were 0.16 for Interpersonal Emotional Competence and 0.22 for Intrapersonal Emotional Competence, indicating low to moderate indirect associations. Finally, emotional competence had an indirect association with post-traumatic growth through adaptive coping, with a significant indirect association of both Interpersonal Emotional Competence (Estimate = 0.32, 95% CI [0.18, 0.47], *p* < 0.001) and Intrapersonal Emotional Competence (Estimate = 0.35, 95% CI [0.23, 0.47], *p* < 0.001) on post-traumatic growth. The standardized estimates of Interpersonal Emotional Competence and Intrapersonal Emotional Competence were 0.25 and 0.35, respectively, pointing toward moderate indirect associations, suggesting that emotional competence relates to post-traumatic growth indirectly through adaptive coping strategies.

Regarding the explanatory power of this model, emotional competence was able to explain 48% of the variance of adaptive coping. On the other hand, 22% of the variance of wellbeing was explained by the direct association of adaptive coping, as well as the indirect effect of emotional competences. In the case of PTGI, the amount of explained variance by the variables in the model was higher (i.e., 53%).

## Discussion

The main objective of the study was to examine whether adaptive coping strategies statistically accounted for the cross-sectional associations between trait EI and positive psychological outcomes, specifically psychological wellbeing and PTG, among Lebanese youth. The findings provided strong support for the proposed theory-driven indirect-associations model. Intrapersonal and interpersonal emotional competences were positively associated with adaptive coping strategies, which in turn were positively associated with psychological wellbeing and PTG.

As hypothesized, emotional competence was positively associated with adaptive coping strategies (H1), suggesting that students with higher levels of awareness and regulation of their own emotions have a higher chance of engaging in healthy and constructive coping mechanisms. This is concordant with previous literature indicating that emotional competence contributes to the bolstering of individuals’ internal resources to adaptively cope with stress (Chaaya et al., 2025). Previous research has also shown that high trait EI individuals tend to experience greater wellbeing, with emotional regulation and cognitive reappraisal proposed as explanatory processes (Peña-Sarrionandia et al., 2015). This connection is further supported by adaptive coping behaviors, as those with higher trait EI are more likely to engage in coping strategies that alleviate distress and promote wellbeing by enhancing perceived control and competence (Du Plessis, 2023; Augusto-Landa et al., 2024). Thus, individuals with higher emotional competence may be better equipped to manage stress within the given situational challenges (Mohamed et al., 2025). This is especially relevant in Lebanese youth, who frequently experience sociopolitical instability, economic challenges, and intergenerational, collectivistic trauma (El Khoury-Malhame et al., 2024).

Supporting H2 and H3, adaptive coping was significantly associated with both psychological wellbeing and PTG, which indicates that individuals who engage in adaptive coping are more likely to experience higher levels of wellbeing and growth. These findings align with prior research suggesting that a healthy way of coping can play a role in enhancing emotional wellbeing and facilitating personal growth following adversity (Nik Jaafar et al., 2021; El Khoury-Malhame et al., 2024). Additionally, recent studies also identify specific adaptive coping strategies, such as seeking social support and having high levels of mature religiosity, as protective factors among Lebanese youth facing instability (Mahfoud et al., 2023; El Khoury-Malhame et al., 2024). Taken together, these findings underscore the importance of adaptive coping as a possible pathway through which emotional competence may translate into greater psychological wellbeing and post-traumatic growth.

The positive zero-order association between WHO-5 and PTGI was reduced to a non-significant residual correlation in the SEM model, indicating that little covariance remained after their shared association with adaptive coping was modeled. This non-significant result is plausible given that the WHO-5 assesses wellbeing over the preceding 2 weeks, whereas the PTGI-SF assesses perceived positive change resulting from coping with adversity. Post-traumatic growth can coexist with distress and does not necessarily imply a high level of current positive emotion or wellbeing. In its functional definition framework, growth is perceived as generated by distress rather than following its resolution (Tedeschi and Calhoun, 2004). These interpretations are in line with meta-analytic evidence showing that posttraumatic growth is only weakly and inconsistently linked to indices of psychological well-being (Helgeson et al., 2006).

The significant indirect-effects findings (H4, H5) underscore the role of adaptive coping as a possible key mechanism linking emotional competences to wellbeing and PTG. This indirect association was stronger with PTG than with wellbeing, suggesting that emotional competence may play a more significant role in facilitating personal growth than overall wellbeing. However, overall, these findings suggest that enhancing emotional competence may indirectly improve both wellbeing and growth by strengthening coping abilities. This supports existing literature suggesting that trait EI facilitates better emotional regulation and coping with distress, ultimately contributing to positive psychological outcomes (e.g., Sanchez-Ruiz et al., 2021b).

These findings (H4, H5) highlight adaptive coping as a potential associational variable linking emotional competences with wellbeing and PTG. Rather than merely buffering distress, adaptive coping appears to channel emotional competence into active psychological adjustment. The indirect associations were stronger for PTG than for wellbeing, suggesting that emotional competences, through adaptive coping, may be particularly relevant for transformative rather than hedonic outcomes. This pattern supports the notion that youth reporting high trait EI are better able to reinterpret adversity in constructive terms, engage in goal-directed action, and extract growth-oriented meaning from hardship (Sanchez-Ruiz et al., 2021b). In this sense, the indirect effect goes beyond emotional regulation as it may reflect a deeper, emotional adaptation process through which individuals not only manage stress but also learn and grow from it.

More specifically, the results point toward the importance of fostering adaptive coping skills, such as active coping, planning, acceptance, and positive reframing in interventions aimed at enhancing emotional competences and fostering personal strength, growth, and appreciation of life. For Lebanese youth, who are navigating chronic sociopolitical and economic instability, developing such competences may be particularly protective. Consistent with recent findings (Sfeir et al., 2023), individuals high in trait EI and adaptive coping report healthier lifestyles and greater wellbeing during crises. Accordingly, interventions that target emotional competences in this context may not only mitigate distress but also cultivate growth, resilience, and appreciation of life, supporting young people in turning adversity into a catalyst for personal development.

### Limitations, implications, and future recommendations

Despite promising findings from the present study, some limitations must be acknowledged. First, the cross-sectional associational variable design precludes any causal inferences; longitudinal and experimental designs are needed to establish causality. Second, the reliance on self-report measures introduces the potential for social desirability and mono-method biases, which may have influenced the accuracy of the data. Additionally, while the indirect-effects associations were statistically supported, the proportion of explained variance in wellbeing and PTG was modest. This suggests that other psychosocial resources not included in the model, such as social support networks, religious or spiritual coping, or broader resilience factors, could be playing a substantial role in shaping outcomes.

The internal consistency of the PEC scores was modest in this sample. These coefficients suggest significant measurement error, which could reduce the accuracy of emotional competence estimates and weaken associations with adaptive coping and outcomes. While the latent-variable SEM models account for indicator-specific measurement error, they do not eliminate concerns arising from limited score reliability. Consequently, PEC-related effect sizes should be interpreted with caution and replicated using more reliable measures of trait emotional intelligence.

In addition, the sample comprised adolescents from public schools in Lebanon, thus limiting generalizability to other socio-economic and educational contexts. Moreover, given Lebanon’s sociocultural context, collective and community-oriented coping strategies might represent key protective processes that remain unaccounted for in the present framework. The cultural and geopolitical specificity of Lebanon, marked by ongoing instability, may also influence how emotional competence, coping, and PTG unfold. Future research should therefore test these mechanisms in more diverse samples and cross-cultural settings, both stable and unstable, to clarify which protective pathways are universal and which are context-dependent, since emotional functioning and coping are shaped by cultural and contextual challenges.

Additionally, trauma exposure was not categorized systematically according to the type, severity, timing or cumulative burden of potentially traumatic events. Instead, participants’ experiences were examined in relation to distressing events occurring within the broader Lebanese sociopolitical context. Consequently, although the findings offer insight into the potential role of emotional competences among adolescents living in a context of chronic adversity, they should be interpreted with caution in relation to post-traumatic growth. Future studies with Lebanese adolescents should include trauma assessments and a detailed trauma typology. This would enable researchers to determine more precisely whether perceived growth occurs specifically in response to traumatic stress, and to examine whether distinct patterns of psychological growth and adjustment are associated with different trauma profiles.

Despite the limitations, findings from this study have relevant implications, both theoretical and practical. Theoretically, the study strengthens the growing body of evidence supporting the critical role of emotional competences as predictors of adaptive coping and positive psychological outcomes among youth. The study extends trait EI literature by examining this indirect-effects model in a trauma-exposed Middle Eastern context. Practically, the results underscore the urgent need to integrate EI training into school-based psychosocial programs, especially in regions affected by chronic instability, such as Lebanon. In this context, the YEY initiative presents a promising approach to equip adolescents with the emotional tools necessary for wellbeing and growth amid ongoing adversity (Sanchez-Ruiz et al., 2023). Moreover, policymakers, educators, and practitioners can leverage these insights to promote EI development as a preventive strategy against mental health deterioration in youth populations.

Although the present study assessed emotional competencies using the Profile of Emotional Competence, future research could examine whether the observed relationships generalize across alternative conceptualizations and measures of emotional competence (e.g., Rodríguez Hernández et al., 2025), thereby providing a broader understanding of emotional functioning and its contribution to wellbeing and post-traumatic growth. Furthermore, expanding the sample to include diverse educational (e.g., private schools, vocational training institutes) and geographic settings within and possibly beyond Lebanon enhances generalizability. Given the moderate effect sizes, alternative coping strategies (e.g., meaning-centered coping), and broader protective resources such as social support and resilience (Li et al., 2022; Mehraban et al., 2022), all of which may play a significant role in shaping positive psychological outcomes. Investigating other associational variables, such as character strengths and virtues, may also offer deeper insight into the pathways to positive psychological outcomes (Ros-Morente et al., 2018).

## Data Availability

The datasets used and/or analyzed during the current study are available from the corresponding author upon reasonable request.

## References

[B1] Alghamdi M. (2020). Cross-cultural validation and psychometric properties of the Arabic brief COPE in Saudi population. *Med. J. Malaysia* 75:502. 32918417

[B2] Al-Khalil Z. M. El Sheikh W. G. Lababidi G. H. Shehayeb E. O. Ghanime P. M. Talih F. R. et al. (2025). Impact of socioeconomic and political stressors on mental health: A cross-sectional study on university students in Lebanon. *BMC Med. Educ*. 25:91. 10.1186/s12909-025-06701-1 39827125 PMC11743072

[B3] Aouani H. Slimani M. Bragazzi N. L. Hamrouni S. Elloumi M. (2019). A preliminary validation of the Arabic version of the &quot; Profile of emotional competence&quot; questionnaire among Tunisian adolescent athletes and nonathletes: Insights and implications for sports psychology. *Psychol. Res. Behav. Manag*. 12, 155–167. 10.2147/PRBM.S188481 30936757 PMC6422408

[B4] Arora J. Kumar V. V. (2023). Coping as a mediator in the relationship between trait emotional intelligence and adolescent stress. *Int. J. Adolesc. Youth* 28, 2292070. 10.1080/02673843.2023.2292070

[B5] Augusto-Landa J. M. García-Martínez I. León S. P. (2024). Analysis of the effect of emotional intelligence and coping strategies on the anxiety, stress and depression levels of university students. *Psychol. Rep*. 127, 1751–1770. 10.1177/00332941221144603 36471637

[B6] Bech P. Østergaard S. D. (2005). “WHO-5 well-being index,” in *Assessment of Quality of Life in Clinical Practice*, ed. van Slooten R. M. M. (Denmark: Nordic Psychological Association), 173–177.

[B7] Bellizzi K. M. Blank T. O. (2006). Predicting posttraumatic growth in breast cancer survivors. *Health Psychol*. 25, 47–56. 10.1037/0278-6133.25.1.47 16448297

[B8] Brasseur S. Grégoire J. Bourdu R. Mikolajczak M. (2013). The profile of emotional competence (PEC): Development and validation of a self-reported measure that fits dimensions of emotional competence theory. *PLoS One* 8:e62635. 10.1371/journal.pone.0062635 23671616 PMC3646043

[B9] Buchlmayer A. Snyder L. Fox C. Schleider J. Smith A. Jans L. et al. (2023). Impact of a single-session growth mindset intervention on adolescent lebanese and syrian refugees. *Eur. J. Psychol. Open* 82, 62–63.

[B10] Cann A. Calhoun L. G. Tedeschi R. G. (2010). The posttraumatic growth inventory short form (PTGI-SF): Validation of the PTGI-SF in a sample of breast cancer survivors. *J. Trauma. Stress* 23, 588–590. 10.1002/jts.20580 20979218

[B11] Cannavò M. Sanchez-Ruiz M. J. Barberis N. (2024). Structural links from trait emotional intelligence to life satisfaction and depressive symptoms in women with breast cancer: Post-traumatic responses as mediators. *Arch. Womens Ment. Health* 27, 383–392. 10.1007/s00737-024-01438-0 38294494 PMC11116199

[B12] Carver C. S. (1997). You want to measure coping but your protocol’s too long: Consider the brief COPE. *Int. J. Behav. Med*. 4, 92–100. 10.1207/s15327558ijbm0401_6 16250744

[B13] Chaaya R. Sfeir M. Khoury S. E. Malhab S. B. Khoury-Malhame M. E. (2025). Adaptive versus maladaptive coping strategies: Insight from Lebanese young adults navigating multiple crises. *BMC Public Health*. 25:1464. 10.1186/s12889-025-22608-4 40259293 PMC12010593

[B14] Compas B. E. Jaser S. S. Bettis A. H. Watson K. H. Gruhn M. A. Dunbar J. P. et al. (2017). Coping, emotion regulation, and psychopathology in childhood and adolescence: A meta-analysis and narrative review. *Psychol. Bull*. 143, 939–991. 10.1037/bul0000110 28616996 PMC7310319

[B15] Costa A. Faria L. (2025). Do students’ mindsets about emotional intelligence change over secondary school? Developmental paths in adolescence. *Soc. Psychol. Educ.* 28, 68. 10.1007/s11218-025-10031-x

[B16] Doumit R. Hallit S. Chaaya R. Zgheib P. El Hajj S. Sanchez-Ruiz M. J. et al. (2026). Psychometric properties of the Arabic translation of the PTGI-SF 10-item scale in adolescents. *Eur. J. Psychotraumatol*. 17:2648457. 10.1080/20008066.2026.2648457 42089355 PMC13151763

[B17] Doumit R. Hallit S. Sanchez-Ruiz M. J. El Khoury-Malhame M. (2025). Psychometric validation of an Arabic version of the WHO-5 wellbeing index among lebanese adolescents. *PLoS One* 20:e0317644. 10.1371/journal.pone.0317644 40674291 PMC12270098

[B18] Du Plessis M. (2023). Trait emotional intelligence and flourishing: The mediating role of positive coping behaviour. *SA J. Indust. Psychol.* 49:2063. 10.4102/sajip.v49i0.2063

[B19] El Khoury-Malhame M. Bou Malhab S. Chaaya R. Sfeir M. El Khoury S. (2024). Coping during socio-political uncertainty. *Front. Psychiatry*. 14:1267603. 10.3389/fpsyt.2023.1267603 38318483 PMC10839968

[B20] Fekih-Romdhane F. Cherif W. Alhuwailah A. Fawaz M. Shuwiekh H. A. M. Helmy M. et al. (2025). Cross-country validation of the Arabic version of the WHO-5 well-being index in non-clinical young adults from six Arab countries. *Glob. Ment. Health*. 12:e95. 10.1017/gmh.2025.10051 40895825 PMC12394027

[B21] Fekih-Romdhane F. Fawaz M. Hallit R. Sawma T. Obeid S. Hallit S. (2024). Psychometric properties of an Arabic translation of the 10-item Connor-Davidson resilience scale (CD-RISC-10), the 8- and 10-item post-traumatic growth inventory-short form (PTGI-SF) scales. *PLoS One* 19:e0293079. 10.1371/journal.pone.0293079 38166051 PMC10760825

[B22] Franke G. R. (2010). “Multicollinearity,” in *Wiley International Encyclopedia of Marketing*, eds Sheth J. Malhotra N. (Chichester: John Wiley & Sons), 10.1002/9781444316568.wiem02066

[B23] Gardner A. A. Lambert C. A. (2019). Examining the interplay of self-esteem, trait-emotional intelligence, and age with depression across adolescence. *J. Adolesc*. 71, 162–166. 10.1016/j.adolescence.2019.01.008 30738220

[B24] Helgeson V. S. Reynolds K. A. Tomich P. L. (2006). A meta-analytic review of benefit finding and growth. *J. Consult. Clin. Psychol*. 74, 797–816. 10.1037/0022-006X.74.5.797 17032085

[B25] Hooper D. Coughlan J. Mullen M. (2008). Structural equation modelling: Guidelines for determining model fit. *Electron. J. Bus. Res. Methods* 6, 53–60.

[B26] Hu L. Bentler P. M. (1999). Cutoff criteria for fit indexes in covariance structure analysis: Conventional criteria versus new alternatives. *Struct. Equ. Modeling* 6, 1–55. 10.1080/10705519909540118

[B27] Johnston R. Jones K. Manley D. (2018). Confounding and collinearity in regression analysis: A cautionary tale and an alternative procedure, illustrated by studies of British voting behaviour. *Qual. Quant*. 52, 1957–1976. 10.1007/s11135-017-0584-6 29937587 PMC5993839

[B28] Khalaf T. Tadros N. Petrides K. V. Mavroveli S. Sanchez-Ruiz M. J. (2026). Investigating the discriminant, criterion, and incremental validity of the English version of the TEIQue in Lebanon. *Curr. Psychol.* 45:343. 10.1007/s12144-025-08704-z

[B29] Kline R. B. (2023). *Principles and Practice of Structural Equation Modeling.* New York, NY: Guilford Publications.

[B30] Li N. Cheng H. Lv L. (2022). The relationship between posttraumatic growth, self-efficacy, and social support in burn patients: A cross-sectional study. *Burns* 48, 1626–1631. 10.1016/j.burns.2022.08.009 36116994

[B31] Mahfoud D. Fawaz M. Obeid S. Hallit S. (2023). The co-moderating effect of social support and religiosity in the association between psychological distress and coping strategies in a sample of lebanese adults. *BMC Psychol*. 11:61. 10.1186/s40359-023-01102-9 36879325 PMC9987350

[B32] Mancini G. Özal Z. Biolcati R. Trombini E. Petrides K. V. (2024). Trait emotional intelligence and adolescent psychological well-being: A systematic review. *Int. J. Adolesc. Youth* 29:2292057. 10.1080/02673843.2023.2292057

[B33] Mehraban E. D. Farhangi A. Abolghasemi S. (2022). The relationship between mental health and perceived social support with the post-traumatic growth model through the mediating role of coping strategies in COVID-19 recovered patients. *J. Shahrekord Univ. Med. Sci.* 24, 182–188.

[B34] Meyer B. (2001). Coping with severe mental illness: Relations of the Brief COPE with symptoms, functioning, and well-being. *J. Psychopathol. Behav. Assess.* 23, 265–277. 10.1023/A:1012731520781

[B35] Mohamed M. G. Al-Yafeai T. M. Adam S. Hossain M. M. Ravi R. K. Jalo F. M. et al. (2025). The significance of emotional intelligence in academic stress, resilience, and safe transition from high school to university: An SEM analysis among northern emirati university students. *Glob. Transit.* 7, 109–117. 10.1016/j.glt.2025.02.003

[B36] Mugavero M. J. Raper J. L. Reif S. Whetten K. Leserman J. Thielman N. M. et al. (2009). Overload: Impact of incident stressful events on antiretroviral medication adherence and virologic failure in a longitudinal, multisite human immunodeficiency virus cohort study. *Psychosom. Med*. 71, 920–926. 10.1097/PSY.0b013e3181bfe8d2 19875634 PMC3691857

[B37] Nasr R. Rahman A. A. Haddad C. Nasr N. Karam J. Hayek J. et al. (2024). The impact of financial stress on student wellbeing in Lebanese higher education. *BMC Public Health* 24:1809. 10.1186/s12889-024-19312-0 38971735 PMC11227159

[B38] Nik Jaafar N. R. Abd Hamid N. Hamdan N. A. Rajandram R. K. Mahadevan R. Mohamad Yunus M. R. et al. (2021). Posttraumatic growth and coping strategies among patients with head and neck cancer: Do approach coping and avoidant coping predict posttraumatic growth over time? *Front. Psychol*. 12:716674. 10.3389/fpsyg.2021.716674 34764904 PMC8576436

[B39] Nozaki Y. Puente-Martínez A. Mikolajczak M. (2019). Evaluating the higher-order structure of the profile of emotional competence (PEC): Confirmatory factor analysis and bayesian structural equation modeling. *PLoS One* 14:e0225070. 10.1371/journal.pone.0225070 31725760 PMC6855477

[B40] O’Donnell P. (2024). Neurobiological underpinnings of adolescent susceptibility to stress. *Biol. Psychiatry Glob. Open Sci*. 4:100364. 10.1016/j.bpsgos.2024.100364 39713127 PMC11662265

[B41] Park C. L. (2010). Making sense of the meaning literature: An integrative review of meaning making and its effects on adjustment to stressful life events. *Psychol. Bull*. 136, 257–301. 10.1037/a0018301 20192563

[B42] Peña-Sarrionandia A. Mikolajczak M. Gross J. J. (2015). Integrating emotion regulation and emotional intelligence traditions: A meta-analysis. *Front. Psychol*. 6:160. 10.3389/fpsyg.2015.00160 25759676 PMC4338658

[B43] Petrides K. V. Mikolajczak M. Mavroveli S. Sanchez-Ruiz M. J. Furnham A. Pérez-González J. C. (2016). Developments in trait emotional intelligence research. *Emot. Rev.* 8, 335–341. 10.1177/1754073916650493

[B44] Petrides K. V. Pita R. Kokkinaki F. (2007). The location of trait emotional intelligence in personality factor space. *Br. J. Psychol*. 98, 273–289. 10.1348/000712606X120618 17456273

[B45] Rodríguez Hernández A. F. Hernández-Jorge C. M. Delgado Hernández J. (2025). Beyond emotional intelligence: Validation of a model of emotional competence applied to teachers. *Educ. Sci.* 15:1157. 10.3390/educsci15091157

[B46] Ros-Morente A. Mora C. A. Nadal C. T. Belled A. B. Berenguer N. J. (2018). An examination of the relationship between emotional intelligence, positive affect and character strengths and virtues. *Anales Psicología Annals Psychol.* 34, 63–67. 10.6018/analesps.34.1.262891

[B47] Sánchez-Álvarez N. Extremera N. Fernández-Berrocal P. (2016). The relation between emotional intelligence and subjective well-being: A meta-analytic investigation. *J. Posit. Psychol.* 11, 276–285. 10.1080/17439760.2015.1058968

[B48] Sanchez-Ruiz M. J. El Khoury M. Doumit R. Alhassanieh M. Chaaya R. Zgheib P. et al. (2023). “Increasing emotional intelligence through yes to emotions in youth (YEY): Evidence from its implementation in Lebanese public schools,” in *40th Biennial Conference of the International Society for the Study of Individual Differences Book of Abstracts*, eds Papageorgiou K. Wilson P. Aitken Schermer J. (Northern Ireland: Queen’s University Belfast), 195–196.

[B49] Sanchez-Ruiz M. J. El-Jor C. Abi Kharma J. Bassil M. Zeeni N. (2019). Personality, emotion-related variables, and media pressure predict eating disorders via disordered eating in Lebanese university students. *Eat. Weight Disord*. 24, 313–322. 10.1007/s40519-017-0387-8 28421473

[B50] Sanchez-Ruiz M. J. El-Hajj S. Gimenez-Lozano J. M. Hernández-Saguar A. El Khoury-Malhame M. Doumit R. et al. (2026). The effectiveness of an emotional intelligence intervention on Lebanese adolescent mental health: A mixed-method study with emotional diary integration. *Br. J. Dev. Psychol.* 1–26. 10.1111/bjdp.70061 42533834 PMC13648305

[B51] Sanchez-Ruiz M. J. Khalaf T. Tadros N. Nauffal D. Nader J. Diab R. et al. (2024). Positive affect and self-care mediate the relationship between trait emotional intelligence and academic engagement in Lebanese undergraduates: Lessons learned from an online setting. *Int. J. Psychol*. 59, 932–941. 10.1002/ijop.13215 38978300

[B52] Sanchez-Ruiz M. J. Mavroveli S. Petrides K. V. (2021a). The trait emotional intelligence questionnaire in lebanon and the UK: A comparison of the psychometric properties in each country. *Int. J. Psychol*. 56, 304–313. 10.1002/ijop.12711 33073867

[B53] Sanchez-Ruiz M. J. Tadros N. Khalaf T. Ego V. Eisenbeck N. Carreno D. F. et al. (2021b). Trait emotional intelligence and wellbeing during the pandemic: The mediating role of meaning-centered coping. *Front. Psychol*. 12:648401. 10.3389/fpsyg.2021.648401 34054650 PMC8155707

[B54] Sfeir M. Akel M. Hallit S. Obeid S. (2023). Factors associated with general well-being among Lebanese adults: The role of emotional intelligence, fear of COVID, healthy lifestyle, coping strategies (avoidance and approach). *Curr. Psychol.* 42, 17465–17474. 10.1007/s12144-021-02549-y 35018085 PMC8736324

[B55] Shakespeare-Finch J. Lurie-Beck J. (2014). A meta-analytic clarification of the relationship between posttraumatic growth and symptoms of posttraumatic distress disorder. *J. Anxiety. Disord*. 28, 223–229. 10.1016/j.janxdis.2013.10.005 24291397

[B56] Sibai A. M. Chaaya M. Tohme R. A. Mahfoud Z. Al-Amin H. (2009). Validation of the Arabic version of the 5-item WHO well being index in elderly population. *Int. J. Geriatr. Psychiatry*. 24, 106–107. 10.1002/gps.2079 18615780

[B57] Taku K. Cann A. Calhoun L. G. Tedeschi R. G. (2008). The factor structure of the posttraumatic growth inventory: A comparison of five models using confirmatory factor analysis. *J. Trauma. Stress*. 21, 158–164. 10.1002/jts.20305 18404631

[B58] Tedeschi R. G. Calhoun L. G. (2004). Posttraumatic growth: Conceptual foundations and empirical evidence. *Psychological. Inquiry.* 15, 1–18. 10.1207/s15327965pli1501_01 42531577

[B59] Tuck D. Patlamazoglou L. (2019). The relationship between traumatic stress, emotional intelligence, and posttraumatic growth. *J. Loss. Trauma.* 24, 721–735. 10.1080/15325024.2019.1621543

[B60] Veronese G. Pepe A. (2019). Using the posttraumatic growth inventory–short form with Palestinian helpers living in conflict areas. *Meas. Eval. Couns. Dev.* 52, 207–221. 10.1080/07481756.2018.1547618

[B61] Zhylin M. Malysh V. Mendelo V. Potapiuk L. Halahan V. (2024). The impact of emotional intelligence on coping strategies for psychological trauma. *Environ. Soc. Psychol.* 9:6145. 10.59429/esp.v9i7.6145

