# Supplementary material for: From trait emotional intelligence to wellbeing and growth: the role of adaptive coping during hardship
Source: Front Psychol. 2026 Sep 15;17:1924156. doi: 10.3389/fpsyg.2026.1924156 (PMC13619407; doi:10.3389/fpsyg.2026.1924156)
Supplement: Supplementary file 1 [file Supplementary_file_1.docx]

| Supplementary Material  Table A1 |  |  |
| --- | --- | --- |
| Modified scales |  |  |
| Posttraumatic Growth Inventory - Short Form (PTGI-SF) |  |  |
| Items | 10-item PTGI-SF | 8-item PTGI-SF |
| PTGI_1 - I changed my priorities about what is important in life | X |  |
| PTGI_2 - I learned a great deal about how wonderful people are | X | X |
| PTGI_3 - I have a greater appreciation for the value of my own life | X | X |
| PTGI_4 - I have a better understanding of spiritual matters | X | X |
| PTGI_5 - I established a new path for my life | X |  |
| PTGI_6 - I have a greater sense of closeness with others | X | X |
| PTGI_7 - I discovered that I’m stronger than I thought I was | X | X |
| PTGI_8 - I have a stronger religious faith | X | X |
| PTGI_9 - I am able to do better things with my life | X | X |
| PTGI_10 - I know better that I can handle difficulties | X | X |
| Brief Coping Orientation to Problems Experienced Inventory (Brief-COPE) |  |  |
| Items | 20-item Brief-COPE | Adaptive coping Brief-COPE |
| COPE_1 - I've been concentrating my efforts on doing something about the situation I'm in | X | X |
| COPE_2 - I've been saying to myself "this isn't real" | X | X |
| COPE_3 - I've been using alcohol or other drugs to make myself feel better | X |  |
| COPE_4 - I've been getting emotional support from others | X |  |
| COPE_5 - I've been giving up trying to deal with it | X |  |
| COPE_6 - I've been taking action to try to make the situation better | X | X |
| COPE_7 - I've been refusing to believe that it has happened | X |  |
| COPE_8 - I’ve been getting help and advice from other people | X |  |
| COPE_9 - I've been using alcohol or other drugs to help me get through it | X |  |
| COPE_10 - I've been trying to see it in a different light, to make it seem more positive | X | X |
| COPE_11 - I’ve been criticizing myself | X |  |
| COPE_12 - I've been trying to come up with a strategy about what to do | X | X |
| COPE_13 - I've been getting comfort and understanding from someone | X |  |
| COPE_14 - I've been giving up the attempt to cope | X |  |
| COPE_15 - I've been looking for something good in what is happening | X | X |
| COPE_16 - I've been accepting the reality of the fact that it has happened | X | X |
| COPE_17 - I’ve been trying to get advice or help from other people about what to do | X |  |
| COPE_18 - I've been learning to live with it | X | X |
| COPE_19 - I've been thinking hard about what steps to take | X | X |
| COPE_20 - I’ve been blaming myself for things that happened | X |  |

*Note.* This table provides a comparison between the original and modified versions of the Posttraumatic Growth Inventory–Short Form (PTGI-SF) and the Brief Coping Orientation to Problems Experienced Inventory (Brief-COPE) used in the present study and identifies the items retained and omitted from the original scales.
